# New insight into podocyte slit diaphragm, a therapeutic target of proteinuria

**DOI:** 10.1007/s10157-020-01854-3

**Published:** 2020-02-04

**Authors:** Hiroshi Kawachi, Yoshiyasu Fukusumi

**Affiliations:** Department of Cell Biology, Kidney Research Center, Niigata University Graduate School of Medical and Dental Sciences, 1-757 Asahimachi-dori, Chuo-ku, Niigata, 951-8510 Japan

**Keywords:** Slit diaphragm, Podocyte, Nephrin, Proteinuria, Thrapeutic target

## Abstract

Dysfunction of slit diaphragm, a cell–cell junction of glomerular podocytes, is involved in the development of proteinuria in several glomerular diseases. Slit diaphragm should be a target of a novel therapy for proteinuria. Nephrin, NEPH1, P-cadherin, FAT, and ephrin-B1 were reported to be extracellular components forming a molecular sieve of the slit diaphragm. Several cytoplasmic proteins such as ZO-1, podocin, CD2AP, MAGI proteins and Par-complex molecules were identified as scaffold proteins linking the slit diaphragm to the cytoskeleton. In this article, new insights into these molecules and the pathogenic roles of the dysfunction of these molecules were introduced. The slit diaphragm functions not only as a barrier but also as a signaling platform transfer the signal to the inside of the cell. For maintaining the slit diaphragm function properly, the phosphorylation level of nephrin is strictly regulated. The recent studies on the signaling pathway from nephrin, NEPH1, and ephrin-B1 were reviewed. Although the mechanism regulating the function of the slit diaphragm had remained unclear, recent studies revealed TRPC6 and angiotensin II-regulating mechanisms play a critical role in regulating the barrier function of the slit diaphragm. In this review, recent investigations on the regulation of the slit diaphragm function were reviewed, and a strategy for the establishment of a novel therapy for proteinuria was proposed.

## Introduction

Proteinuria is one of the most important symptoms of kidney diseases and is reported to be one of the most important risk factors of stroke and cardiovascular diseases [1]. However, the etiology, pathogenesis, and clinical significance of proteinuria were not fully understood yet. The development of more effective selective therapy for proteinuria is awaited. Glomerular proteinuria is associated with pathological damage of the glomerular filtration barrier, which is composed of three layers: glomerular endothelial cells, glomerular basement membrane (GBM), and glomerular visceral epithelial cells (podocytes). The studies in the past two decades revealed that the third layer, podocyte functions as the final barrier [2, 3]. It is now widely accepted that slit diaphragm, a cell–cell junction of podocytes, plays a critical role in preventing the leak of plasma proteins into primary urine and that dysfunction of the slit diaphragm is involved in the development of proteinuria in several glomerular diseases. The functional molecules of the slit diaphragm could be novel therapeutic targets for proteinuria.

The authors have previously reviewed the role of the slit diaphragm [4, 5]. In this review, first, we will overview a unique, specialized property of the slit diaphragm, review new insight into the structural and functional properties of the slit diaphragm, and discuss the future line of the investigations for establishing a novel therapy for proteinuria.

## Overview: slit diaphragm, a specialized cell–cell junction of podocyte, shares common characteristics with synapse

Foot processes of podocytes cover the outer side of GBM. The neighboring foot processes were derived from different cell bodies, and they were connected by a continuous membrane-like structure, which is called “slit diaphragm”. The early study with electron microscopy showed that the slit diaphragm exhibited a zipper-like substructure with alternating, periodic cross-bridges extending from the opposite podocyte plasma membranes [6]. Arakawa demonstrated that neighboring foot processes were interdigitated with the scanning electron microscopic analysis [7]. Developmental analyses showed the slit diaphragms appear during the capillary loop stage and gradually replace tight junctions [8, 9]. The slit diaphragm is reported to be a specialized adherens junction, because it contains typical adherens junction proteins such as P-cadherin, β-catenin [10], and FAT [11], while Farquhar’s group reported that slit diaphragm is a highly specialized variant of a tight junction, because the slit diaphragm contains several tight junction proteins including ZO-1, JAM-A, occludin, and cingulin [12].

Podocyte shares common characteristics with neuron: both of them are final differentiated cells, which have no ability of proliferation, and they possess unique processes and share common functional molecules such as synaptopodin [13], densin [14], drebrin [15], and dendrin [16]. These molecules are restrictively expressed in podocyte foot processes and neuronal dendric spines. Slit diaphragm connects foot processes of podocytes. Synapse is a structure between axon terminal and dendrite of neurons. Both are highly specialized cell–cell junctions connecting unique processes. Rastaldi et al. reported that Rab3, a synaptic vesicle surface protein, is expressed at the slit diaphragm and that the vesicles locate at the edge of the foot process [17]. Synaptic vesicle-associated protein 2B (SV2B) is also expressed at slit diaphragm [18]. A recent study showed that the deletion of SV2B results in the mislocalization of the slit diaphragm components including nephrin and CD2AP and that several synaptic vesicle-associated molecules including SNARE molecules were expressed in podocyte [19]. Soda et al. reported that dynamin, synaptojanin, and endophilin, which are functional partners in synaptic vesicle recycling, are essential for the formation and maintenance of the podocyte foot process structure [20]. It is reported that neurexin-1, a presynaptic adhesion molecule, is expressed in podocyte [21], and neurexin-1 interacts with CD2AP and SV2B. It is plausible that dysfunction of these neuron-associated molecules in podocyte is involved in the development of slit diaphragm dysfunction and that the pharmacological reagent targeting these molecules could be candidates for a novel therapy protecting podocyte.

## Extracellular components forming a molecular sieve of the slit diaphragm

The first molecule identified as an extracellular component of the slit diaphragm is nephrin [22]. Nephrin is accepted to be the main body of the extracellular portion of the slit diaphragm. Nephrin has a long extracellular domain containing eight Ig-like modules and a single fibronectin type III module. Ruotsalainen et al. proposed that nephrin molecules extending from two adjacent foot processes are likely to interact with each other in the slit through homophilic interactions [23], as has been shown for other Ig cell adhesion molecules such as N-CAM [24], C-CAM [25], and L1 [26]. Nephrin was identified as a product of the mutated gene in patients with Finnish-type nephrotic syndrome [22], and it was demonstrated that the antibody against nephrin is capable of inducing massive proteinuria [27–29]. Based on these findings, nephrin is accepted to be a key molecule forming a molecular sieve of the slit diaphragm. Downregulation of nephrin is observed in experimental nephrotic models [28, 30–32] and in clinical cases of several types of glomerular disease such as minimal change disease [33], membranous nephropathy, membranoproliferative glomerulonephritis, IgA nephropathy, lupus nephritis [34], diabetic kidney disease [35, 36], and preeclampsia [37]. These studies indicated that nephrin dysfunction is one of the common pathogenic mechanism of proteinuria in human glomerular diseases.

Another critical molecule of the extracellular components is NEPH1. NEPH1 was identified as a nephrin-related protein by the gene trapping screen [38]. NEPH1, a transmembrane protein, contains five extracellular immunoglobulin-like domains [38]. NEPH1 interacted with nephrin in a cis form [39]. Glomeruli of NEPH1 knock-out mice showed the effacement of podocyte foot processes and proteinuria, and all mice died at 3–8 weeks of age [38], which indicates that NEPH1 is also essential for maintaining the barrier function of the slit diaphragm. A recent study using high-resolution ultrastructural imaging showed that slit diaphragm is multilayered and that the NEPH1 molecule spanning is in the lower part of the junction, closer to GBM with a width of 23 nm, while nephrin contributes to the apical region of the slit diaphragm with a width of 45 nm [40]. The study also reported that nephrin is not required to build a functional filtration barrier in birds, and discussed that NEPH1 plays a critical role in birds.

Some of other transmembrane proteins were reported to be accumulated at the slit diaphragm. Reiser et al. reported that P-cadherin, a member of cadherin superfamily, was localized at the slit diaphragm [10]. Cadherin forms homophilic Ca^2+^-dependent cell–cell adhesion. FAT, a large transmembrane protein of 34 tandem cadherin-like repeats, was localized at the slit diaphragm and was costained with nephrin [11]. FAT KO mice showed severe proteinuria, which indicates that FAT is also essential for maintaining the filtration barrier of the slit diaphragm [41]. It was also reported that neurexin [21] and ephrin-B1 [42] are accumulated at the slit diaphragm. Neurexin is a presynaptic adhesion molecule and is known to have multiple splicing variants. It was shown that a unique variant of neurexin is expressed in the podocyte. Immunoprecipitation assay with rat glomerular lysate showed that neurexin interacted with CD2AP, a cytoplasmic molecule binding to nephrin [21]. Ephrin-B1 is a protein of Eph-ephrin family, which has many biological functions in several types of tissues [43]. Ephrin-B1 interacts with nephrin via their extracellular portions. The podocyte selective ephrin-B1 KO mice displayed mild but significant proteinuria [44], indicating that ephrin-B1 participates in maintaining the barrier function of the slit diaphragm. However, the molecular components forming a sieve structure of the extracellular domain of the slit diaphragm are not well clarified yet.

## Multiple scaffold proteins participate in the link of slit diaphragm to the cytoskeleton

**Fig. 1:**
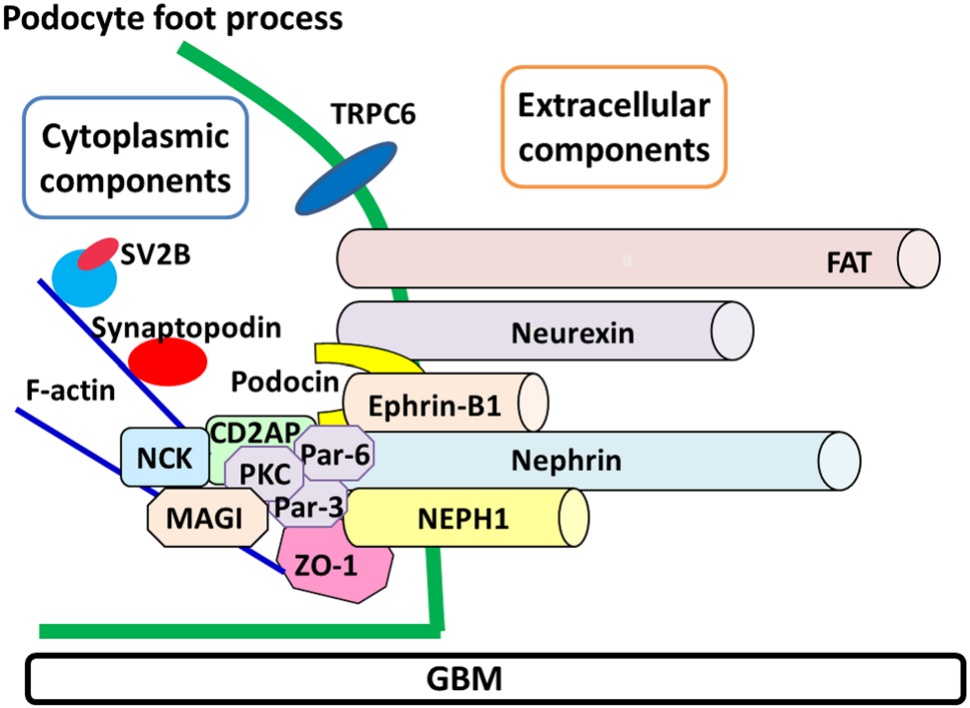
Schematic diagram of the slit diaphragm. Nephrin, NEPH1, ephrin-B1, FAT, and neurexin form the extracellular region of the slit diaphragm. The extracellular components of the slit diaphragm interact with the scaffold proteins, podocin, CD2AP, ZO-1, Nck, MAGI, and Par-complex molecules (Par-3, Par-6, aPKC)

Multiple cytoplasmic proteins linking the membrane proteins at the slit diaphragm to the cytoskeleton were identified. Schematic interaction of the scaffold proteins and transmembrane proteins at the slit diaphragm was shown in Fig. 1. ZO-1 (zonula occludens-1) was reported to be localized at the cytoplasm just below the cell surface at the insertion point of slit diaphragm [45]. ZO-1 is the first molecule identified as a component of the slit diaphragm. ZO-1 was originally identified as a component of tight junction and belongs to a family of the membrane–associated guanylate kinase (MAGUK) molecules with PDZ domains [46]. ZO-1 directly interacts with NEPH1 by binding of the first PDZ domain of ZO-1 to the C-terminus PDZ binding motif of NEPH1 [47]. The interaction between NEPH1 and ZO-1 was lost in response to the ischemic injury leading to podocyte foot process effacement and proteinuria [48]. The recent study intending novel drug development showed that isodesmosine, a naturally occurring compound, can protect podocyte from injury by stabilizing the ZO-1-NEPH1 complex [49]. These reports suggested that the interaction of ZO-1 and NEPH1 is essential for maintaining the slit diaphragm structure, and the interaction could be a target for a drug.

Podocin is identified as a product of a gene mutated in familial steroid-resistant nephrotic syndrome [50]. Since podocin has a single transmembrane region and both N- and C-terminus of podocin are localized at cytoplasm, podocin is considered to have a hairpin-like structure [51]. Podocin interacts with nephrin, NEPH1 and CD2AP [52–54]. The expression and the localization of podocin are altered in several types of acquired glomerular diseases such as FSGS [55], lupus nephritis [56] and IgA nephropathy [57], and in experimental nephrotic models [58]. Podocin is a mammalian homolog of the *C. elegans* stomatin family protein Mec-2. Mec-2 is recruited to the putative mechanosensory complex in *C. elegans* touch sensory neurons [59]. Podocin interacts with TRPC6, one of the key regulators of slit diaphragm function. Knockdown of podocin markedly increased stretch-evoked activation of TRPC6. It is also reported that podocin deficiency results in Ca^2+^ overload in foot processes [60]. Podocin regulates the barrier function of the slit diaphragm by acting as a switch to determine the preferred mode of TRPC6 activation.

CD2AP, an 80 kDa protein, has been shown to interact with nephrin [61]. CD2AP was identified as an SH3-containing protein that binds to the cytoplasmic domain of CD2, a membrane protein on T cell and natural killer cell. CD2AP anchors nephrin to the cytoskeleton, since CD2AP has an actin-binding site at the N-terminus. Mice lacking CD2AP exhibit morphological alterations such as loss of foot process, severe proteinuria [62]. Kim et al. reported that two human patients with focal segmental sclerosis had a mutation predicted to ablate the expression of one CD2AP allele [63]. It has been shown that lack of CD2AP leads to the increased expression of TGF-β and promotes the TGF-β-induced apoptosis [64]. The study indicated that CD2AP regulates the survival of podocyte by regulating the expression of TGF-β. It is also reported that dendrin binds CD2AP and nephrin at the slit diaphragm [16]. The report showed that dendrin relocates to the nucleus of injured podocytes and that nuclear dendrin modulates TGF-β-induced apoptosis. Following the report, Yaddanapudi et al. reported that loss or downregulation of CD2AP allowed for an increase in TGF-β signaling and the translocation of dendrin from the slit diaphragm into the nucleus. Dendrin is a transcription factor specifically promoting the expression of cytosolic CatL. Cytosolic CatL, in turn, drove the reorganization of the actin cytoskeleton. Then, it was concluded that CD2AP functions as the gatekeeper of the podocyte TGF-β response through its regulation of cytosolic CatL expression [65]. Very recently, Tossidou et al. reported that CD2AP is a phosphorylation target of receptor tyrosine kinases stimulated by VEGF-A [66]. They demonstrated that phosphorylation of tyrosine at position Y10 of the SH3-1 domain of CD2AP could change the affinity of CD2AP to nephrin and is indispensable for CD2AP function.

MAGI proteins (MAGI-1, MAGI-2, MAGI-3) belong to the MAGUK family function as molecular scaffolds, coordinating signaling complexes by linking cell surface receptors to the cytoskeleton. MAGI-1 interacts with junctional adhesion molecule 4 (JAM4), and both MAGI-1 and JAM-4 are expressed in podocytes [67]. Immunoelectron microscopy shows that the localization of MAGI-1 is restricted to the slit diaphragm, whereas JAM4 is distributed at the slit diaphragm and on apical membranes. The in vitro interaction assay showed that MAGI-1 binds nephrin via the middle PDZ domains of MAGI-1 and the carboxyl terminus nephrin [68, 69]. It is understood that MAGI-1 forms a tripartite complex with nephrin and JAM4 at the slit diaphragm [68]. The studies with nephrotic models showed that MAGI-1 and JAM 4 are downregulated in the proteinuric states [68, 70]. MAGI-2 is also expressed in podocyte and directly binds the carboxyl terminus of nephrin. Shirata et al. reported that podocyte-specific conditional MAGI-2-knockout (MAGI-2-CKO) mice exhibited slit diaphragm disruption, morphologic abnormalities of foot processes, and podocyte apoptosis leading to podocyte loss [71]. MAGI-2 interacts with dendrin and plays a role in retaining it at the slit diaphragm. In MAGI-2 CKO mice, dendrin is translocated from the slit diaphragm to the nucleus, and podocyte apoptosis is promoted. Thereby the lack of MAGI-2 in podocyte results in FSGS.

The partitioning-defective (Par)-complex (Par-3/Par-6/aPKC) is understood to be a central player in regulating cell polarity in several cell types. Hartleben et al. reported that Par-3 and aPKC are expressed at podocyte slit diaphragm and NEPH1-nephrin complex binds to the Par-complex [72]. Recently, Takamura et al. demonstrated that Par-3 binds to nephrin, and Par-6 binds to ephrin-B1, another transmembrane protein at slit diaphragm [73]. The mice administered with a dominant-negative aPKC construct showed significant proteinuria, and the loss of foot process architecture was detected in the isolated glomeruli treated with an inhibitor of aPKC. These observations clearly showed that the NEPH1–Nephrin–Par complex is essential for the maintenance of the barrier function of the slit diaphragm. The selective depletion of aPKCλ/ι in mouse podocyte results in slit diaphragm displacement, foot process effacement, proteinuria, and renal failure [74, 75]. It is known that aPKC has two isoforms: aPKCλ/ι and aPKCζ. The double aPKCλ/ι and aPKCζ knockout in podocyte results in severe proteinuria and perinatal death [76]. The developmental study with neonatal mice by Huber et al. showed that Par polarity complex translocated from apical to basal during glomerulogenesis and that the translocation proceeds slit diaphragm formation [76, 77]. These findings suggested that aPKC signaling is a fundamental mechanism for the development and maintenance of podocyte slit diaphragm.

Synaptopodin plays a critical role in maintaining podocyte function and is reported to be a target of cyclosporine, an immunosuppresive agent which is used for patients with nephrotic syndrome [78]. Synaptopodin was originally identified as an actin-associated protein of podocytes [13]. Synaptopodin interacts with α-actinin-4 and regulates its actin-bundling activity at foot processes [79]. Synaptopodin interacts with CD2AP at the slit diaphragm [80, 81]. Yu et al. reported that synaptopodin affects the localization and function of TRPC6 [82]. Synaptopodin participates in the regulation of the slit diaphragm function through regulating the TRPC6 function.

## Slit diaphragm functions as a signaling platform

The slit diaphragm functions not only as a barrier but also as a signaling platform transfer the signal to the inside of the cell. Nephrin, a key transmembrane protein of the extracellular domain of the slit diaphragm, has several tyrosine residues. The tyrosine residues of nephrin can be phosphorylated by Src family kinases, including Src, Fyn, Lyn, and Yes [83–85]. The tyrosine residues inducing signaling pathways were divided into two groups. The first group is Y1114 (YEES), Y1138/9 (YYRS) (human numbering system). Phosphorylation of the first group tyrosines induces the binding to p85/PI3K [86, 87]. The second group tyrosine is YDxV motif, which includes Y1176 (YDEV), Y1193 (YDEV), and Y1217 (YDQV). Phosphorylation of tyrosines of the second group promotes the recruitment of NCK, an SH2/SH3 containing adaptor protein [88, 89]. It is also reported that nephrin phosphorylated at the tyrosine residue of this group linked to another SH2/SH3 containing protein PLB-γ1 [90, 91].

There is still a lack of consensus on whether phosphorylation of tyrosines of nephrin is associated with promotion or protection of podocyte injury, and it is understood that there are site-specific differences in phosphorylation in baseline, injury, and recovery. Tyrosine phosphorylation of nephrin induces two forms of the structure of actin in culture. One form is lamellipodia, in which two-dimensional actin mesh is observed. Lamellipodia is sometimes associated with foot process effacement of podocyte seen in the pathogenic state in vivo and is mediated by the phosphorylation of tyrosines of the first group [85, 86]. Another form is growth of actin polymer (production of actin tail at nephrin), which is considered to be associated with the stabilization of the foot process and the maintenance of the slit diaphragm structure. The production of the actin tail is basically mediated by the phosphorylation of the second group tyrosines [89]. The reduced phosphorylation level of the second group tyrosines was detected in human glomerular diseases such as minimal change disease [92], membranous nephropathy [93]. The recent study shows that the knock-in mice of which three tyrosine residues of Y1191, Y1208, and Y1232 of mouse numbering system, which corresponds to the second group tyrosine residues in human, were converted to phenylalanine, developed progressive proteinuria accompanied by structural changes [94]. The results indicated that phosphorylation of these tyrosines is required for stabilization of podocyte morphology. Phosphorylation of tyrosines of nephrin is negatively regulated by several tyrosine phosphatases. Alterations in levels of tyrosine phosphatases are also detected in several pathogenic states. Protein tyrosine phosphatase 1B, which can directly dephosphorylate tyrosines of the second group, is upregulated in rat puromycin aminonucleoside nephropathy [95]. SH2 domain-containing phosphatase 1, which de-phosphorylates tyrosines of the second group, is increased in models of diabetic nephropathy [96]. C1-Ten, which is recently identified as nephrin tyrosine phosphatase targeting tyrosines of the first group (Y1114 and Y1138) is also upregulated in diabetic nephropathy [97]. These recent findings showed that a reduction of nephrin phosphorylation level is involved in the development of podocyte injury in diabetic nephropathy. It is also reported that the phosphorylation status of nephrin regulated its own endocytosis. The studies on the endocytosis mechanism of nephrin will be described in the next section. A recent study demonstrated that nephrin signaling results in integrin β1 activation. The finding implied that nephrin-mediated signal regulates podocyte attachment to glomerular basement membrane [98].

NEPH1 also can transduce outside-in signals. Four tyrosine residues (Y637, Y638, Y716, Y719) of NEPH1 were identified that became phosphorylated. NEPH1 phosphorylation results in the recruitment of Grb2. Fyn is necessary for NEPH1-Grb2 interaction. The interaction results in actin polymerization [99]. Phosphorylated NEPH1 and nephrin coordinate distinct signaling pathway through binding to different SH2 domain proteins Grb2 and NCK. NEPH1 is phosphorylated in several injured podocyte models [48, 100, 101]*.* Since inhibiting NEPH1 phosphorylation protects podocyte from injury, it is assumed that inhibiting NEPH1 signaling is therapeutically significant in preventing podocyte damage [48].

**Fig. 2:**
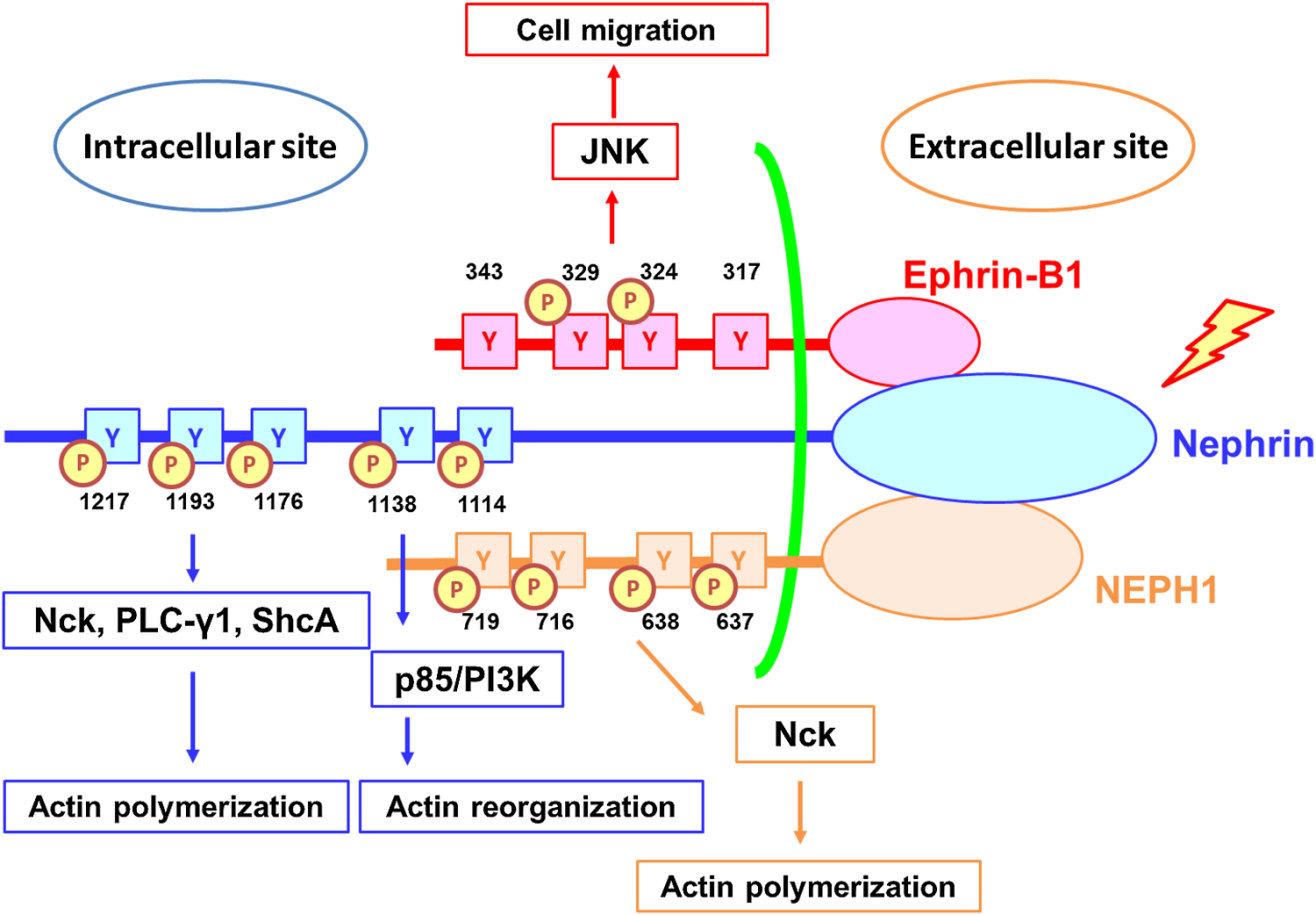
Roles of tyrosine phosphorylation of nephrin, NEPH1, and ephrin-B1. Phosphorylation (P) of tyrosine residues (Y) of nephrin and NEPH1 regulates actin polymerization and reorganization. Ephrin-B1 phosphorylation regulates JNK signaling

Ephrin-B1 at slit diaphragm also functions as a signaling molecule. The nephrin-binding ephrin-B1 at slit diaphragm transfers the signals nephrin detected to the inside of the cell via different ways from nephrin-mediated signaling [44]. If ephrin-B1 is phosphorylated, nephrin and Par-6 were dissociated from ephrin-B1 [44, 73]. Phosphorylation of ephrin-B1 promoted mobility of podocyte through activation of JNK. It is estimated that the promoted mobility participates in podocyte injury. Regulation of phospho-status of ephrin-B1 could be a therapeutic target. Roles of phosphorylation of tyrosine residues of nephrin, NEPH1, and ephrin-B1 are summarized in Fig. 2.

## Mechanisms regulating the slit diaphragm function

The dynamics of podocyte morphology and the function of the slit diaphragm are regulated by calcium signaling. TRPC5 and TRPC6 channels have been identified in podocyte, and they play a central role in regulating calcium influx and affect the reorganization of the actin cytoskeleton of podocyte [102]. TRPC6 couples with RhoA, while TRPC5 couples with Rac. Although TRPC5-mediated signals have been considered to cause podocyte injury leading to cytoskeletal collapse [103], the function of TRPC5 in podocytes is not clearly understood. Winn et al. reported that a mutation of TRPC6, proline to glutamine at position 112 (P112Q), was detected in patients of familiar FSGS [104]. Reiser et al. reported that other TRPC6 mutations R895C and E897K were detected in the patients [105]. They showed that these mutations lead to increased calcium influx. It is understood that the gain-of-function alteration causes podocyte injury leading to FSGS. On the contrary, recently a loss of function type of TRPC6 mutation was found to be associated with human FSGS [106]. The report suggests that unbalanced calcium entry dysregulated by TRPC6 causes podocyte damage. TRPC6 affects not only FSGS but also other diseases with podocyte injury. It is observed that the expression of TRPC6 was increased in diabetic nephropathy [107–109]. TRPC6 interacts with podocin, which suggests that the regulation of calcium signaling mediated by TRPC6 is highly associated with the maintenance of slit diaphragm function.

Inhibiting angiotensin II (AngII) action has been known to be beneficial in many types of kidney diseases. Several studies have suggested that ACEI and AngII type 1 receptor blockade (ARB) have a protective role for slit diaphragm by promoting the expression of nephrin, podocin, and NEPH1 [110–113]. Although the pharmacological mechanism of these drugs is not fully explained yet, it is reported that AngII action affects the slit diaphragm by regulating the TRPC6 function. The study analyzing the mechanism of AngII-induced apoptosis showed that the protein level of TRPC6 was increased markedly in response to Ang II and that the intracellular Ca^2+^ concentration was elevated [114]. The study also showed that if TRPC6 was knocked down with siRNA, Ang II-induced podocyte apoptosis and the transient Ca^2+^ influx were inhibited. The role of TRPC6 in AngII-induced podocyte injury was also demonstrated by in vivo study [115]. The study revealed that TRPC6-deficient mice had significantly less albuminuria. These observations showed that AngII action causes podocyte injury by enhancing the TRPC6 function. Nijenhuis et al. proposed an attractive hypothesis explaining the mechanism of AngII-induced podocyte injury [116]. AngII type 1 receptor stimulation by AngII results in Ca^2+^ influx mediated by TRPC6. Ca^2+^-dependent calcineurin activation leads to activation and nuclear translocation of NFAT, which enhances transcription of NFAT-responsive genes such as TRPC6. A consequent increase in TRPC6 expression at the cell membrane could result in a positive feedback regulatory circuit. The positive feedback mechanism can result in persistent calcineurin activation and promotes the podocyte injury. It is also proposed that calcineurin inhibitor such as cyclosporine ameliorates podocyte injury by blocking the positive circuit. The breaking down of the positive circuit can be a plausible strategy of a novel therapy for protecting podocyte.

Since AT1R is a member of Gq-coupled receptors, the beneficial effect of agents inhibiting the activation of AT1R may be mediated at least in part by inhibition of Gq signaling. Wang et al. reported that constitutive active Gq-alpha subunit promoted podocyte injury by stimulating calcineurin activity, resulting in calcineurin-dependent upregulation of TRPC6 [117]. It is also reported that other G protein-coupled receptors (GPCRs) contribute to kidney injury by activating TRPC6. Roshanravan et al. demonstrated that ATP evoked activation of TRPC6 through G protein-coupled pathway from P2Y receptors [118]. A recent study by Wang et al. demonstrated that activation of group I mGluRs induced TRPC6-dependent Ca^2+^ influx [119]. An alternative treatment strategy might be to target the signaling pathway from GPCRs. Especially, agents inhibiting Gq activation and therapy targeting downstream signaling cascade linked to Gq activation might be useful novel therapy for podocyte injury.

Recently, Verheijden et al. [120] demonstrated that the stimulation of TRPC6-dependent calcium influx increased calpain-1 and calcineurin activity and reduced the expression of a calpain target Talin-1. The study also showed that in kidneys of patients with FSGS, calpain, and calcineurin activity, as well as TRPC6 expression were increased, and the expression of Talin-1 was clearly reduced. Talin-1 links the actin cytoskeleton to integrins, and is critical for podocyte cytoskeletal stability. Very recently, it was reported that for activation of calpain, the physical interaction between TRPC6 and calpain is important and TRPC6 channel activity is independent [121]. It is conceivable that calpain-1 inhibition could be future therapeutic options to treat patients with FSGS.

A slit diaphragm is understood to be a highly dynamic unit. Regular replacement of the slit diaphragm components is necessary for maintaining the integrity of slit diaphragm. However, little is known about the mechanism regulating endocytosis and recycling back to the plasma membrane of the transmembrane proteins of the slit diaphragm. In podocytes two endocytic pathways, clathrin-dependent and clathrin-independent endocytosis, have been identified. Quack et al. reported that β-arrestin mediated clathrin-dependent endocytosis of nephrin [122]**.** The study demonstrated that β -arrestin interacts with nephrin dephosphorylated at Y1193. Phosphorylation of nephrin Y1193 by Fyn enhances the interaction of nephrin with podocin, and prevents the interaction with β -arrestin, and attenuates the β -arrestin-mediated nephrin endocytosis. It is also reported that Fyn-mediated phosphorylation of nephrin promoted nephrin endocytosis via the clathrin-independent pathway [84, 123]. It is plausible that the phosphorylated state shifts nephrin to the different endocytic pathways. Recent report showed that ShcA, a SH2-containing protein, binds to the nephrin phosphorylated at Y1193 and promotes nephrin endocytosis [124]. These observations showed that the phosphorylation status of nephrin Y1193 determines the slit diaphragm integrity. It is proposed that the regulation of the phosphorylation status of nephrin Y1193 could be a strategy for a novel therapy for proteinuria. The study by Soda et al. showed that dynamin participates in the mechanism of nephrin endocytosis [20]. It is reported that activation of Notch signaling induces nephrin internalization via a β -arrestin/dynamin-dependent route [125]. Activation of Notch signaling was detected in podocyte injury with proteinuria of several types of human glomerular disease [126, 127]. Notch activation is considered to function as a molecular switch that triggers irreversible podocyte injury and promotes disease progression in proteinuric glomerulopathy. The therapeutic approach targeting Notch may promote the repair of the glomerular filtration barrier and prevent terminal podocyte injury.

## Future lines of study: a strategy for the establishment of a novel therapy for proteinuria

In this review, we overview the podocyte functional molecules that play a critical role in the maintenance of the barrier function of the slit diaphragm. The molecular structure of the slit diaphragm and the mechanism regulating the function of the slit diaphragm are much more complicated than we thought in the 1990s. Although a lot of studies were piled up, the nature of the slit diaphragm is not fully clarified yet, and the therapy targeting the slit diaphragm molecules is not established yet.

The transmembrane proteins forming a molecular sieve of the slit diaphragm such as nephrin, NEPH1, and ephrin-B1 must be prior targets for a novel therapy for proteinuria. Of course, nephrin should be the most important target; however, some recent reports suggested that the stabilization of NEPH1 is an essential strategy to protect podocyte from serious damages [49]. The authors have been analyzing the expression of the slit diaphragm molecules in several nephrotic models and observed that nephrotic model in which NEPH1 is downregulated from the early phase shows persistent proteinuria and progress to irreversible podocyte injury [32]. However, our knowledge on the mechanism of turnover of NEPH1 is very limited. The transcriptional mechanism and the endocytosis mechanism of NEPH1 should be clarified.

The modifications of signaling pathways from the slit diaphragm should be one of the important strategies for novel therapy for the podocyte injuries. Although the pathological significance of the signaling pathway from nephrin is not precisely understood, several studies showed that the phosphorylation level of each tyrosine residues of nephrin is strictly regulated. It was reported that the phosphorylation level of nephrin is regulated by the activity of the phosphatase such as protein tyrosine phosphatase 1B and C1-Ten [95–97]. These phosphatases may be targets for novel therapy. The study with podocyte-specific conditional knock out mice showed that ephrin-B1 plays a central role in regulating the JNK signaling pathway of podocyte [44]. JNK is a stress-activated kinase and is considered to regulate cell motility of podocyte. It is conceivable that the regulation of the JNK pathway mediated by ephrin-B1 is a rational strategy for protecting podocyte from irreversible injury.

Slit diaphragm is considered to be a variant of tight junction [8, 9, 12, 45], and the transition of slit diaphragm to tight junction appears in several nephrotic conditions [128, 129]. The Par complex is essential for the establishment of cell polarity and the formation of the tight junction. It is reported that the tight link between the Par complex to the slit diaphragm components is critical for maintaining the structure of the slit diaphragm [72, 73]. Reinforcement of the linking between the transmembrane proteins and the Par complex molecules is also a rational strategy.

In this article, we reviewed that the slit diaphragm has common characteristics with synapse. Several synapse-associated molecules are highly expressed in podocytes, and they play a critical role in maintaining the slit diaphragm function. Synaptic vesicle-associated proteins are involved in the maintenance of the slit diaphragm [17–20]. Dendrin, which is originally identified in dendrite of rat neuron, plays a critical role in stabilizing the scaffold proteins of the slit diaphragm [16]. These neuron-associated molecules could be targets for therapy for protecting podocyte. It is expected that some of the drugs and chemical agents developed for neuronal diseases have an effect on podocyte. We established the in vitro system to analyze the expression of these neuronal molecules with cultured podocytes. However, to select the effective agent more sensitive screening system should be established. Recently, the culture condition to induce the cultured podocytes with long arborized processes was reported [130]. The method may help for establishing a better screening system.

Reducing the level of proteinuria is critical for preventing the progression to end-stage kidney. Although proteinuria is accepted to be one of the most important risk factors of stroke and cardiovascular diseases, the causal relationship between proteinuria and these diseases remains to be unclear. We suppose that proteinuria may work as “canary in a coal mine” to predict other disease, because the glomerular capillary wall preventing proteinuria seems to be a very delicate and fragile unit. Therapeutic intervention of proteinuria from the early phase will help to prevent stroke, cardiovascular disease, and some other diseases which have a common pathogenic mechanism with proteinuria. A novel effective therapy for proteinuria targeting slit diaphragm should be established as soon as possible.
